# Action of the Galveston Medical Club, with Reference to the Appointment of State Health Officer

**Published:** 1887-01

**Authors:** 


### ACTION OF GALVESTON MEDICAL CLUB.

To His Excellency, L. S. Ross, Governor of the State of Texas:

At a meeting of the Galveston County Medical Club, held in Galveston on the tenth (10th) day of January, 1887, the following resolutions were unaimously adopted; and it was ordered that a copy of them be sent to his Excellency Gov. Ross, and one to Dr. Swearingen. Also, that copies be furnished each of the Medical Journals, published in Texas, the Galveston News, and Austin Statesman.

Whereas, Galveston is the chief sea port city of Texas, and is consequently the gateway, through which the most dreaded epidemic diseases might, most likely, be introduced into the State; it is natural that we, her resident physicians, who entertain a sincere interest in the welfare of the people of our city and State, should take the initiative in an effort to co-operate with his Excellency Gov. Ross, to prevent their importation, and horrible consequences.

Therefore be it Resolved: 1st. That to further this end, we believe the judicious selection of a State Health Officer is of paramount importance.

2nd. That we recognize the fact that an officer should possess, not only the highest order of professional qualification, but firmness, courage, a keen moral sense, and superior judgment, as well.

3rd. That personal knowledge enables us to bear testimony to the eminent fitness of Dr. R. M. Swearingen of Austin, (the present incumbent) for the position; who embodies not merely the professional and moral qualities enumerated, but an extensive experience of yellow fever, cholera and small pox. Whose heroic services at Holly Springs, Mississippi, during the epidemic of 1878 have given him fame, which should endure as long as benevelence has a name.

4th. That we appreciate the intelligent, manly, humane, and efficient manner in which Dr. Swearingen has performed the responsible duties of the State Health Officer during the past six years; and feel that he has invested it with a dignity which can only be sustained by the brightest and best representative our State can furnish.

5th. That his re-appointment would maintain the feeling of confidence and security his past services have inspired among all classes of our business people; and of satisfaction and pride among the members of the medical profession throughout the States.

6th. That we earnestly request, on behalf of the Physicians of Galveston, to whom our people look for protection against pestilence, that Dr. R. M. Swearingen be reappointed Health Officer of the State of Texas by his Excellency Gov. Ross.

7th. That a copy of these resolutions be sent to his Excellency Gov. Ross.

Committee { J. F. Y. Paine, M. D.<br>C. W. Trueheart, M. D.<br>Edmond Goldman, M. D. }

Hall of Galveston County Medical Club,<br>Galveston, Texas, January 10th, 1887.

